# Supplementary material for: MetaRibo-Seq measures translation in microbiomes
Source: Nat Commun. 2020 Jun 29;11:3268. doi: 10.1038/s41467-020-17081-z (PMC7324362; doi:10.1038/s41467-020-17081-z)
Supplement: Supplementary file 10 — Supplementary Data 7 [file 41467_2020_17081_MOESM10_ESM.zip › File2/Confidence_High_Taxonomy/162017_out.krona.html]

Javascript must be enabled to view this page.

members
magnitude
magnitudeUnassigned
count
unassigned
taxon
rank

162017\_out

2

superkingdom
2
2

phylum
976
2

class
200643
2

2
171549
order

1
2005525
family

375288
genus
1

species
46503

SRS144506\_contig\_number\_32896
1

1
family
1853231

genus
283168

SRS018888\_contig\_number\_7658
1
